# Superior frontal regions reflect the dynamics of task engagement and theta band-related control processes in time-on task effects

**DOI:** 10.1038/s41598-022-04972-y

**Published:** 2022-01-17

**Authors:** Shijing Yu, Moritz Mückschel, Christian Beste

**Affiliations:** 1 Cognitive Neurophysiology, Department of Child and Adolescent Psychiatry, Faculty of Medicine, TU Dresden, Schubertstrasse 42, 01309 Dresden, Germany; 2 University Neuropsychology Centre, Faculty of Medicine, TU Dresden, Dresden, Germany

**Keywords:** Cognitive neuroscience, Cognitive control

## Abstract

Impairment of cognitive performance is often observed in time-on tasks. Theoretical considerations suggest that especially prefrontal cortex cognitive control functions is affected by time-on-task effects, but the role of effort/task engagement is not understood. We examine time-on-task effects in cognitive control on a neurophysiological level using a working-memory modulated response inhibition task and inter-relate prefrontal neuroanatomical region-specific theta-band activity with pupil diameter data using EEG-beamforming approaches. We show that task performance declines with time-on tasks, which was paralleled by a concomitant decreases of task-evoked superior frontal gyrus theta-band activity and a reduction in phasic pupil diameter modulations. A strong relation between cognitive control-related superior frontal theta-band activity and effort/task engagement indexed by phasic pupil diameter modulations was observed in the beginning of the experiment, especially for tasks requiring inhibitory controls and demanding high working memory. This strong relation vanished at the end of the experiment, suggesting a decoupling of cognitive control resources useable for a task and effort invested that characterizes time-on-task effects in prefrontal cortical structures.

## Introduction

Performing tasks for a long time is often accompanied by the feeling of cognitive (mental) fatigue associated with a gradual and cumulative decline of cognitive functions^1–3^. This is not confined to populations already showing fatigue symptoms, but is also evident in otherwise healthy individual. The degree to which sustained task performance leads to the feeling of cognitive fatigue that associated with a gradual and cumulative decline of cognitive functions is often termed time-on-task effect^4^ and many theoretical accounts have been proposed to conceptualize these effects in demanding tasks^5^. One view is that excessive mental effort depletes these mental resources and causes declines in task performance^6–9^. Other accounts have centred on motivation and stress that the repetitive nature leads observers to withdraw effort over time^10–12^.

Kurzban et al.^5^ presented the ‘opportunity cost model’ of time-on-task effect/fatigability. It proposes that when subjects comply with task requirements/instructions, the costs (opportunity costs) of the computational systems required to perform the task are represented. As one gains more experience with a task, the expected benefit to allocate full processing resources and to engage in a task decline. Thus, the representations of costs grow with time-on task. According to Kurzban et al.^5^, this is experienced as fatigue and leads to declines in performance. According to the model, opportunity costs are particularly strong for cognitive functions relying on the prefrontal cortex because this brain structure is involved in various tasks virtually simultaneously. Since computational processes in the prefrontal cortex are subject to simultaneity constraints, this implies that there will be large opportunity costs when performing several tasks depending on prefrontal cortex functions^5^. The functional specialization of prefrontal cortical areas further sharpens these constraints. When engaging in a task, performance in another task strongly depend on the degree to which the two tasks engage similar prefrontal regions^5^. Therefore, specialization contributes to increasing opportunity costs and thus, fatigue^5^. According to Kurzban et al.^5^, the sensation of effort reflects an adaptive problem and its solution—simultaneity and prioritization. Opportunity costs are experienced as effort and reduce task performance^5^. Due to its focus on prefrontal cortex functions, the model by Kurzban et al.^5^ seems particularly suitable to frame investigations of time-on-task effects in cognitive control and executive functions known to depend on prefrontal cortical structures^13–15^. In the current study, we examine time-on-task effects in cognitive control on a neurophysiological level using a working-memory modulated response inhibition task and inter-relate prefrontal neuroanatomical region-specific neurophysiological data focusing on theta-band activity (TBA) with pupil diameter data. The reasons for this are as follows:

According to Kurzban et al.^5^, time-on-task effects (opportunity costs) should be particularly strong when performing several tasks depending on prefrontal cortex functions ‘simultaneously’. Some conceptions suggest that there are three core executive functions e.g.^16,17^: (i) inhibition, (ii) working memory (WM), and (iii) cognitive flexibility^13^. Combining two of these core processes in a task should allow a reliable evaluation of time-on-task processes and how these are reflected on a neurophysiological level. We do so combining working memory processes with inhibitory control processes. Prefrontal cortical networks involved in response inhibition and working memory mechanisms largely overlap^18–23^, which is of relevance according to the simultaneity constraint in the Kurzban account. On a neurophysiological level, response inhibition processes are well-known to depend on TBA^24–33^ and the same is the case for working memory processes^34,35^. Therefore, also the reliance of both processes on TBA contributes to simultaneity constraints stressed in the Kurzban account, making it likely that working memory-modulated response inhibition shows time-on-task effects. Interestingly, it has been shown that some aspects of TBA are sensitive to time-on-task effects^3^. Several studies have shown that especially TBA in the superior frontal gyrus is central to accomplish response inhibition^27,30,36^. Moreover, there is evidence that superior frontal regions also play a role in working memory processes^37–40^. Therefore, we hypothesize that with increasing time on task, behavioral performance should decline, reflected by a concomitant decrease of TBA in superior frontal cortical regions as revealed by applying EEG-beamforming methods.

However, TBA is also relevant considering the aspect of task engagement in the Kurzban account. In the account, task engagement is closely related to opportunity costs, which are experienced as effort and reduce task performance^5^. Studies have shown that TBA during inhibitory control correlates with pupil diameter modulations^20,27^. Generally, the pupil diameter is a viable metric to track mental effort^41–43^ or engagement in the task^44^. Two essential aspects of pupil diameter dynamics can, however, be distinguished: modulations in pupil diameter evoked by task demands have been referred to as phasic pupillary responses^45–49^; the pupil diameter in resting states likely represent tonic pupil activity^19,50,51^. This dissociation refers to the fact that the pupil diameter is at least partly modulated by the norepinephrine (NE) system^42,44,52–55^, known to yield phasic and tonic activity modes^44,54,56^. Task-related, phasic pupil diameter changes have been suggested to reflect phasic norepinephrine responses^45–48^. In line with that, the adaptive gain theory^56^ proposes that phasic NE activity reflects task-related decision processes that become particularly relevant when task-related decision or response selection processes are demanding^56,57^. Stronger phasic NE activity and a larger phasic pupil diameter have been associated with more task engagement^42,44,52–55^. Therefore, task-related (phasic) pupil diameter modulations are suitable to track task engagement and opportunity costs according to the Kurzban model. The phasic pupil diameter should become smaller with time on task. More importantly, there should be inter-relations (correlations) between pupil diameter dynamics across time-on-task and superior frontal TBA modulations across time-on-task. Since the model by Kurzban et al.^5^ assumes that the expected benefit to engage in a task decline with time on task, it is reasonable to hypothesize that the phasic pupil diameter decreases with time. However, no clear-cut hypotheses can be stated for the direction of the correlation between pupil diameter and TBA. Previous data showing positive correlations between the pupil diameter and superior frontal TBA during inhibitory control are based on a completely different task and stimulus specifications^27^. Other data from working memory-modulated response inhibition^20^ did not examine TBA from superior frontal regions. Although there may be positive correlations between superior frontal TBA and the pupil diameter, it is also possible that there are negative correlations (i.e., smaller pupil diameter is related to higher superior frontal TBA). Some studies suggest that (medial) frontal TBA reflects the need to engage in cognitive control^58,59^. When efforts invested in the task are low (indexed by a small phasic pupil diameter), it is even more critical that TBA subserving cognitive control is high (indexed by stronger TBA). However, considering the pattern of significant correlations between superior frontal TBA and pupil diameter, we hypothesize that the correlation changes by increasing time on task. According to Kurzban et al.^5^, the expected benefit to allocate full cognitive control resources to the task declines. This suggests that with time on task, the pupil diameter data (indexing effort invested in the task) and the strength of TBA (indexing the degree of cognitive control useable for the task) should become unrelated. Therefore, it is reasonable to hypothesize the correlation between pupil diameter and TBA becomes weaker with time on task.

## Results

### Questionnaire data

The Fatigue Scale for Motor and Cognitive Functions (FSMC) data revealed an average cognitive score of 16.22 ± 4.70 and an average motor score of 16.63 ± 4.24. Penner et al.^71^ indicated that a cognitive or motor score lower than 22 shows no cognitive or motor fatigue while a cognitive or motor score between 22 to 28 shows mild fatigue. Individual cognitive scores of FSMC showed that *N* = 4 subjects showed mild fatigue (all ≤ 25), and the rest showed no cognitive fatigue, and *N* = 3 subjects showed mild motor fatigue (all ≤ 27). All of the *N* = 27 subjects had a Beck’s Depression Inventory(BDI) score lower than 10 (mean: 2.22 ± 2.06), which shows that participants revealed no depressive symptoms^60^.

### Behavioral data

**Figure 1:**
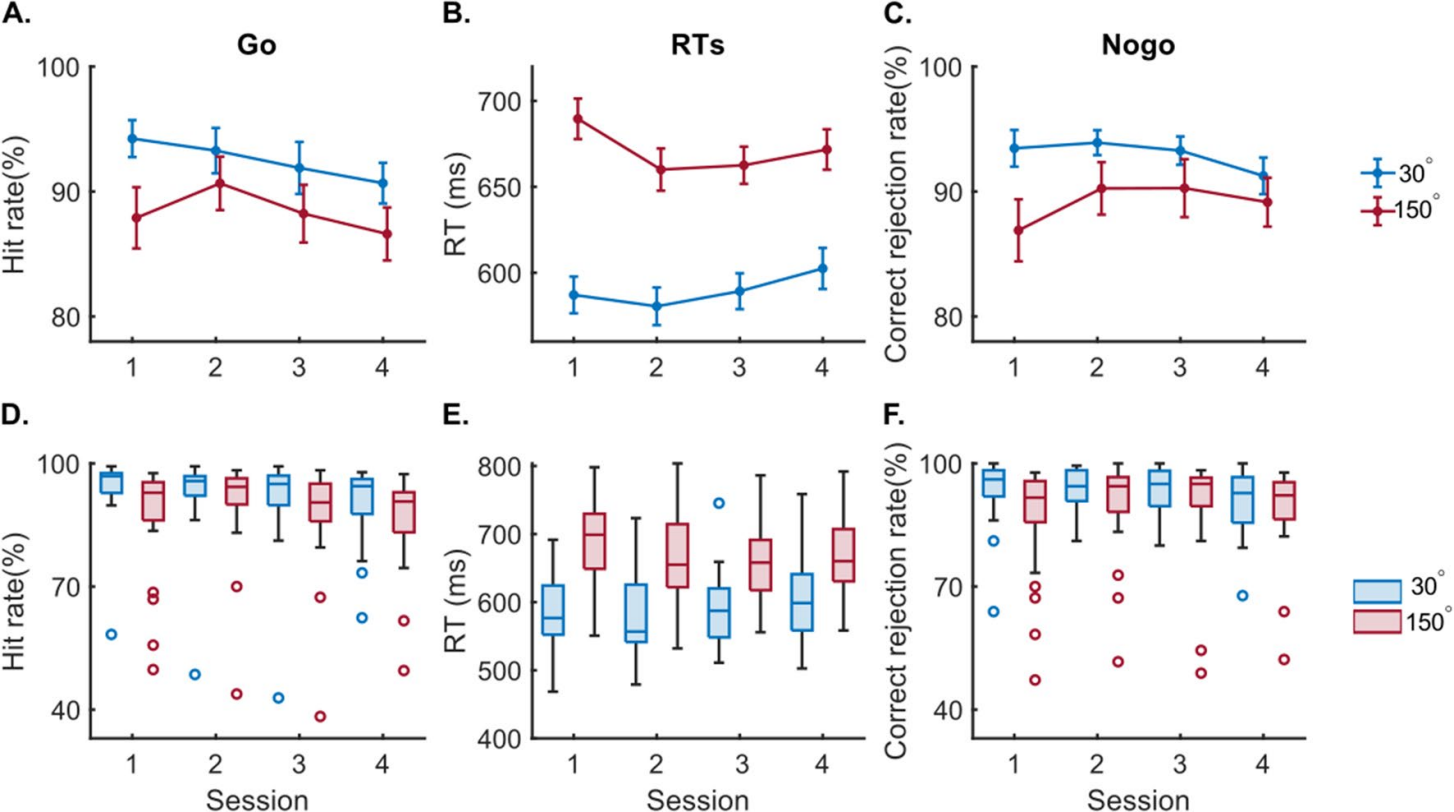
Results of behavioral data. (**A**) shows the rate of correct responses (hit rate) (mean and SEM), (**B**) reaction times (RTs in ms) for each session in Go trials (mean and SEM), (**C**) shows the correct rejection rate (mean and SEM) in Nogo trials for each session. Figure parts (**D**–**F**) show boxplots of the data corresponding to figure parts (**A**–**C**). Each dot represents an outlier. Outliers are defined by default using Matlab ‘boxchart’ function.

Descriptive data of hit rate, reaction time (RT) and correct rejection rate in each condition and session are illustrated in Fig. 1. The means and standard deviations are also shown in Supplementary Table S1.

For Go trials, the repeated measures ANOVA using hit rate (Fig. 1A) revealed a main effect of rotation angle (F(1,26) = 15.33, *p* < 0.001, η^2^ = 0.38) with higher hit rate for 30° condition (92.52 ± 1.68) than 150° (88.34 ± 2.11). A significant main effect of session was evident (F(2.01,52.13) = 4.08, *p* = 0.023, η^2^ = 0.14). Bonferroni-corrected pairwise comparisons showed that hit rate in the S2 session (91.96 ± 1.93) was significantly higher than S3 session (90.06 ± 2.16; *p* = 0.005), the differences between other sessions were not significant (all *p* > 0.059). An interaction between session and rotation angle was also significant (F(1.81,47.03) = 5.06, *p* = 0.012, η^2^ = 0.16). Post-hoc tests were applied for 30° and 150° conditions separately using paired samples t-test. For the 30° condition, significant differences were observed between the first 2 sessions and the last 2 sessions (S1 and S3: t(26) = 2.68, *p* = 0.013, Cohen’s d = 0.515; S1 and S4: t(26) = 3.36, *p* = 0.002, Cohen’s d = 0.647; S2 and S3: t(26) = 2.95, *p* = 0.007, Cohen’s d = 0.567; S2 and S4: t(26) = 2.45, *p* = 0.021, Cohen’s d = 0.472). For the 150°condition, the difference between S2 and other sessions were significant (S1: t(26) = -2.56, *p* = 0.016, Cohen’s d = −0.493; S3: t(26) = 3.67, *p* = 0.001, Cohen’s d = 0.706; S4: (t(26) = 2.79, *p* = 0.010, Cohen’s d = 0.536).

The repeated measures ANOVA using RTs in Go trials (Fig. 1B) revealed a main effect of rotation angle (F(1,26) = 230.63, *p* < 0.001, η^2^ = 0.90), showing that the RTs in 30° condition (589.82 ± 10.12 ms) were shorter than in the 150° condition (670.97 ± 10.50 ms). An interaction effect of “degree × session” (F(2.45,63.69) = 26.40, *p* < 0.001, η^2^ = 0.50) was also observed. For 30° condition, the difference between S4 and the two other sessions (S2 and S3) were significant (S2: t(26) = −2.80, *p* = 0.009, Cohen’s d = −0.540; S3: t(26) = −2.68, *p* = 0.012, Cohen’s d = −0.517). For the 150° condition, the difference between S1 and S2 sessions (t(26) = 6.14, *p* < 0.001, Cohen’s d = 0.681), and between S1 and S3 (t(26) = 3.13, *p* = 0.004, Cohen’s d = 0.603) were significant.

Most important for the current study is the performance on Nogo trials. For correct rejection rate in Nogo trials (Fig. 1C), the repeated measures ANOVA revealed a main effect of rotation angle (F(1,26) = 7.12, *p* = 0.013, η^2^ = 0.22) with a significantly higher correct rejection rate in the 30° condition (92.97 ± 1.12) than in the 150° condition (89.14 ± 2.07). There was no main effect of session (F(2.35,61.12) = 2.21, *p* = 0.11, η^2^ = 0.08). The interaction between session and rotation angle also showed a significant effect (F(2.52,65.59) = 4.04, *p* = 0.015, η^2^ = 0.13). For the post-hoc tests in the 30° condition, significant differences were observed between S4 and all other sessions (S1: t(26) = 2.27, *p* = 0.032, Cohen’s d = 0.437; S2: t(26) = 2.38, *p* = 0.025, Cohen’s d = 0.457; S3: t(26) = 2.50, *p* = 0.019, Cohen’s d = 0.482). In 150°, S1 was different from S2 and S3 sessions (S2: t(26) = −2.72, *p* = 0.011, Cohen’s d = −0.525; S3: t(26) = −2.06, *p* = 0.050, Cohen’s d = −0.396).

### Pupil diameter

**Figure 2:**
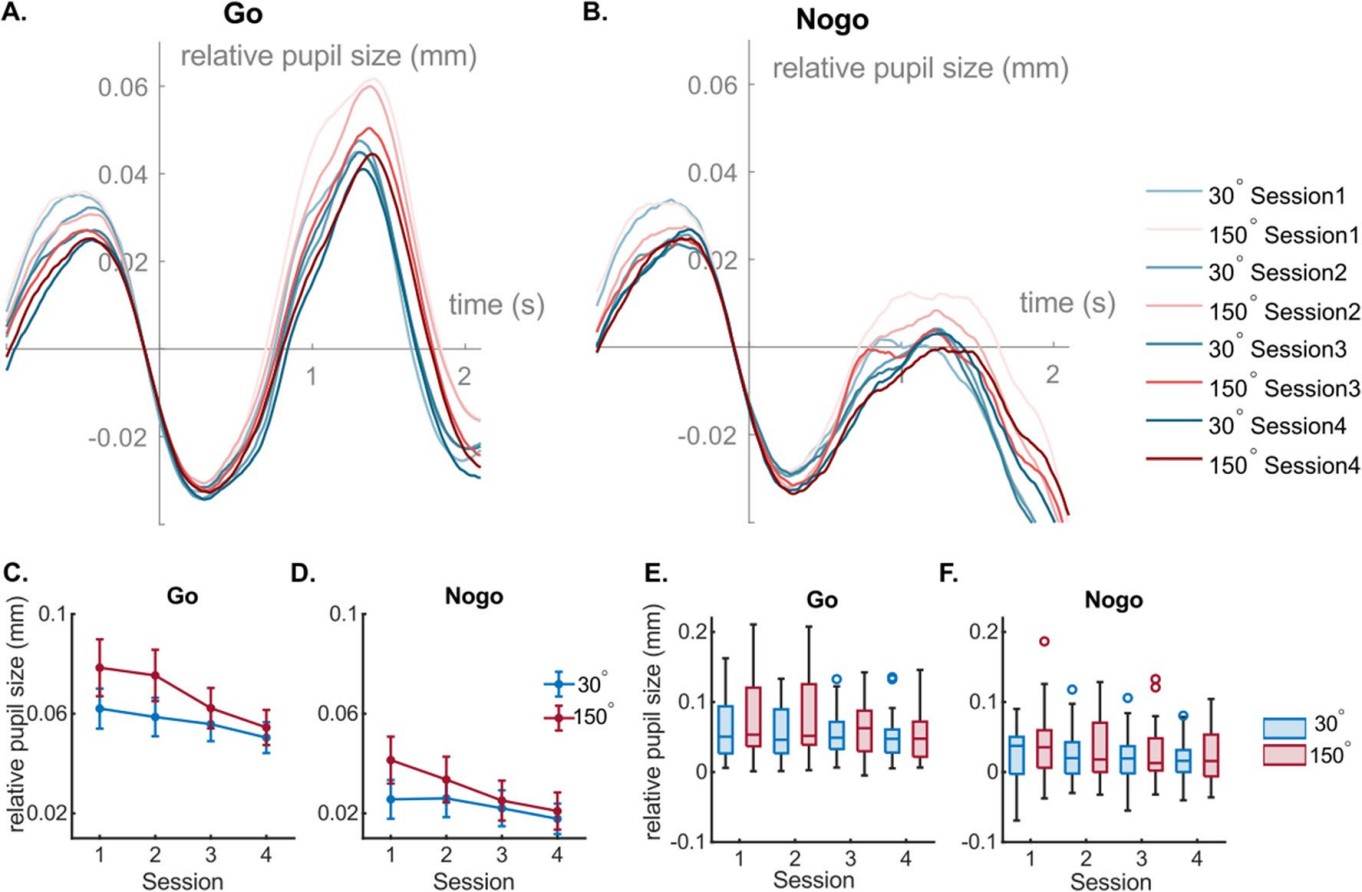
Pupil Diameter Results. (**A**) and (**B**) illustrate the change of average pupil diameter relative to baseline across time for each rotation angle and for Go and Nogo trials. Red and blue colors represent the 30° and the 150° conditions, respectively. Each line in the plots corresponds to a session and a particular condition. Darker colors indicate later sessions. (**C**) and (**D**) present the average and SEM of baseline-corrected amplitudes of the pupil diameter after stimulus presentation in each session for each condition. Figure parts (**E**) and (**F**) show boxplots of the data corresponding to figure parts (**C**) and (**D**). Each dot represents an outlier. The method used for the definition of outliers is the same as for the behavioral data (cf. Fig. 2).

The pupil diameter data consists of two components, task-evoked pupil diameter modulations reflect phasic pupillary responses; the pupil diameter in resting states likely represents tonic pupil activity^19,50,51^. The phasic pupil diameter data are shown in Fig. 2A for Go and 2B for Nogo conditions.

The descriptive data of the corresponding peaks after the onset of the stimulus are illustrated in Fig. 2C, D (average and SEM) as well as Fig. 2E, F (boxplots) (see supplementary Tables 2 and 3 for precise descriptive data). Clear pupil dilations after stimulus presentation were observed in Go trials (Fig. 2A). In Nogo trials, pupil dilations were noticeably slighter, and pupil diameter amplitudes are hardly detected (Fig. 2B). Concerning the peaks of pupil diameter in Go trials (Fig. 2C, E), repeated measures ANOVA revealed a main effect of rotation angle (F(1,26) = 12.62, *p* = 0.001, η^2^ = 0.33) with a larger pupil diameter in the 150° condition (0.068 ± 0.009 mm) than in 30° condition (0.057 ± 0.007 mm). The main effect session was significant (F(1.68,40.76) = 6.10, *p* = 0.008, η^2^ = 0.19), showing a decreasing trend from session S1 (0.070 ± 0.009 mm) to S4 (0.052 ± 0.007 mm). However, Bonferroni-corrected pairwise comparisons revealed no significant difference between any two sessions. The interaction “session × rotation angle” was also significant (F(1,58,41.18) = 4.47, *p* = 0.025, η^2^ = 0.15). The post-hoc tests revealed that in the 150° condition, the pupil diameter amplitudes of the first two sessions were significantly different from the last two sessions (S1 and S3: t(26) = 2.65, *p* = 0.014, Cohen’s d = 0.51; S1 and S4: t(26) = 3.03, *p* = 0.006, Cohen’s d = 0.58; S2 and S3: t(26) = 2.64, *p* = 0.014, Cohen’s d = 0.51; S2 and S4: t(26) = 3.12, *p* = 0.004, Cohen’s d = 0.60). In the 30° condition, no comparison between any sessions was significant (all t > 2.01, *p* > 0.055, Cohen’s d < 0.39).

For Nogo trials (Fig. 2D, F), a main effect was only found for rotation angle (F(1,26) = 5.85, *p* = 0.023, η^2^ = 0.18) that the pupil diameter amplitude were higher in 150° condition (0.030 ± 0.007 mm) than in 30° condition (0.023 ± 0.006 mm). No main effect of session (F(1.93,50.19) = 2.87, *p* = 0.068, η^2^ = 0.10) or interaction of “degree × session” (F(2.61,67.76) = 1.92, *p* = 0.143, η^2^ = 0.07) was observed.

Though the latency of the pupil diameter dilation after stimulus presentation showed an increasing trend across sessions in Supplementary Table S3, the repeated measures ANOVA revealed that only a main effect of rotation angle was observed in both of Go and Nogo conditions (Go: F(1,26) = 6.71, *p* = 0.015, η^2^ = 0.21; Nogo: F(1,26) = 4.52, *p* = 0.043, η^2^ = 0.15), but no other significant main effect or interaction effect was detected (all F < 1.85, *p* > 0.169, η^2^ < 0.07).

In the trial-unrelated resting period (see supplementary Fig. 1), a paired-sample t-test indicated that a significant increase in total pupil diameter from the beginning (3.62 ± 0.33 mm) to the end (3.79 ± 0.51 mm) (t = -3.39, *p* = 0.002, Cohen’s d = −0.665). During the experiment, there was also an increasing trend in pupil size and the one-way ANOVA measures showed that the change was significant (F(7.14,178.44) = 5.23, *p* < 0.001).

### Time–frequency analysis and beamforming

Time–frequency analysis and, more importantly, the beamforming analysis using Fieldtrip relies on a contrast between 2 conditions. It is not possible to model more than 2 conditions in the cluster-based permutation tests for time–frequency data and the calculation of the beamforming sources using Fieldtrip. Therefore, one has to choose 2 conditions when running beamforming analyses to avoid an inflation of type one error due to multiple comparison problems. This is the reason, why, opposed to the task performance behavioral data, only the conditions S1 and S4 were compared, which also showed the most robust effects in the behavioral data. Also, from a theoretical point of view, the time-on-task effect is maximal between S1 and S4. Therefore, we chose this approach for the analysis of TBA and associated functional neuroanatomical sources.

**Figure 3:**
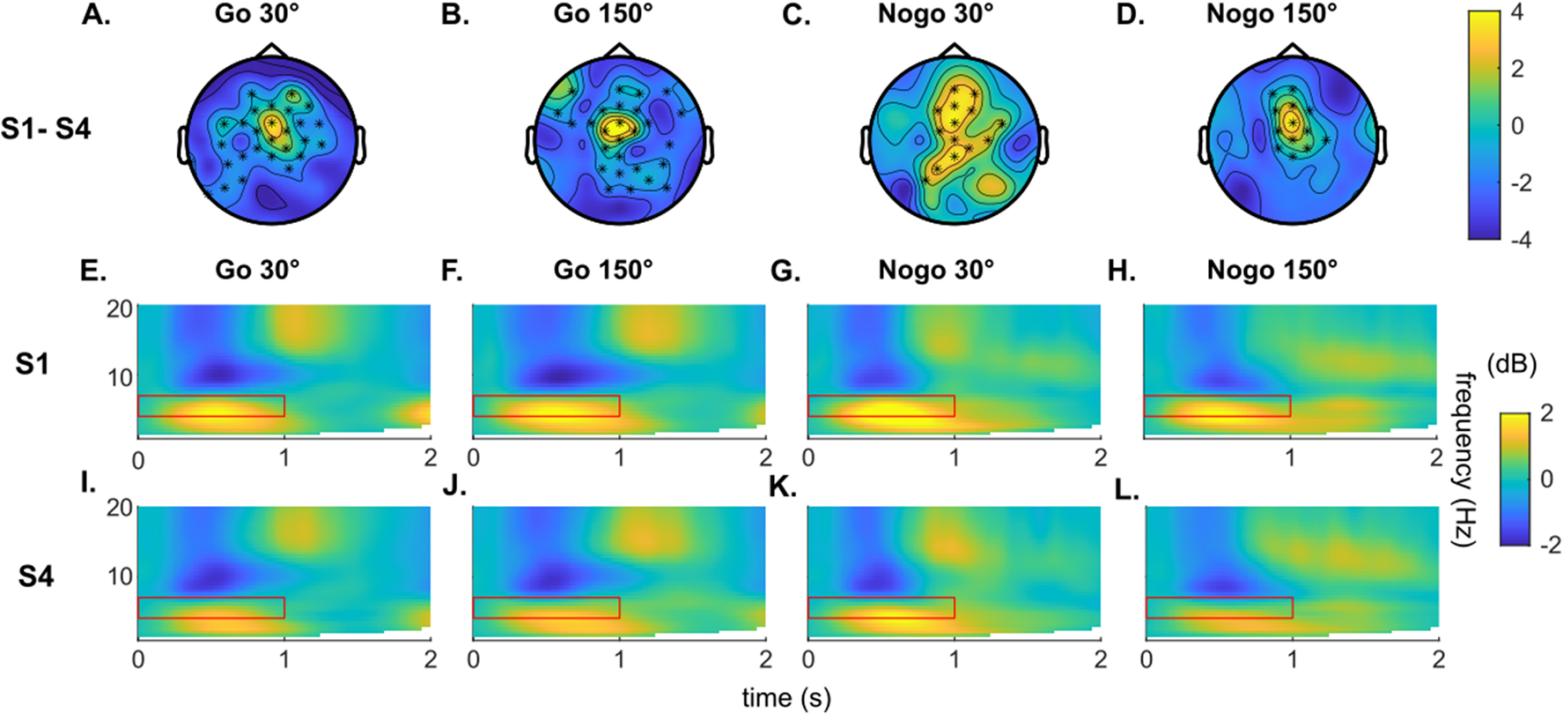
Time–frequency analysis results. (**A**–**D**) illustrate the difference in baseline-corrected theta power (in dB) between S1 and session S4. Each asterisk in the scalp topography plots denotes significant results in the cluster-based permutation test (*p* < .05). The color bar indicated the *t*-values. (**E**–**L**) show the time–frequency plots. The red rectangles mark theta activities (4 ~ 7 Hz) in the time interval of 0 to 1 s.

The cluster-based permutation results revealed a significant change of stimulus-evoked theta activities from session S1 to S4 (*p* < 0.05). The scalp topographies maps in Fig. 3A–D show the electrodes where significant changes were evident.

The electrodes showing significant changes varied in different conditions but still shown some similarity. Most significant differences were found at electrodes ‘Cz’, ‘FCz’, ‘FC1’, ‘Fz’, ‘F1’, ‘CPz’ and ‘F2’ in all conditions. Therefore, we averaged the time–frequency results from these electrodes to show the time–frequency plots (Fig. 3E–L). The figures reveal clear theta activity. Paired-sample t-tests were performed to compare averaged theta band activity between S1 and S4 in the time interval of 0 to 1 s. There was a significant decrease from session S1 to session S4 in baseline-corrected theta activities within 0–1 s (Go 30°: t(26) = 4.41, *p* = 0.004; Go 150° : t(26) = 3.342, *p* = 0.004; Nogo 30°: t(26) = 3.49, *p* = 0.005; Nogo 150°: t(26) = 3.26, *p* = 0.005).

The beamforming procedure showed that the primary source of theta band activity revealing differences between session S1 and session S4 were located in the frontal cortex with the location slightly varying between the different conditions.

**Figure 4:**
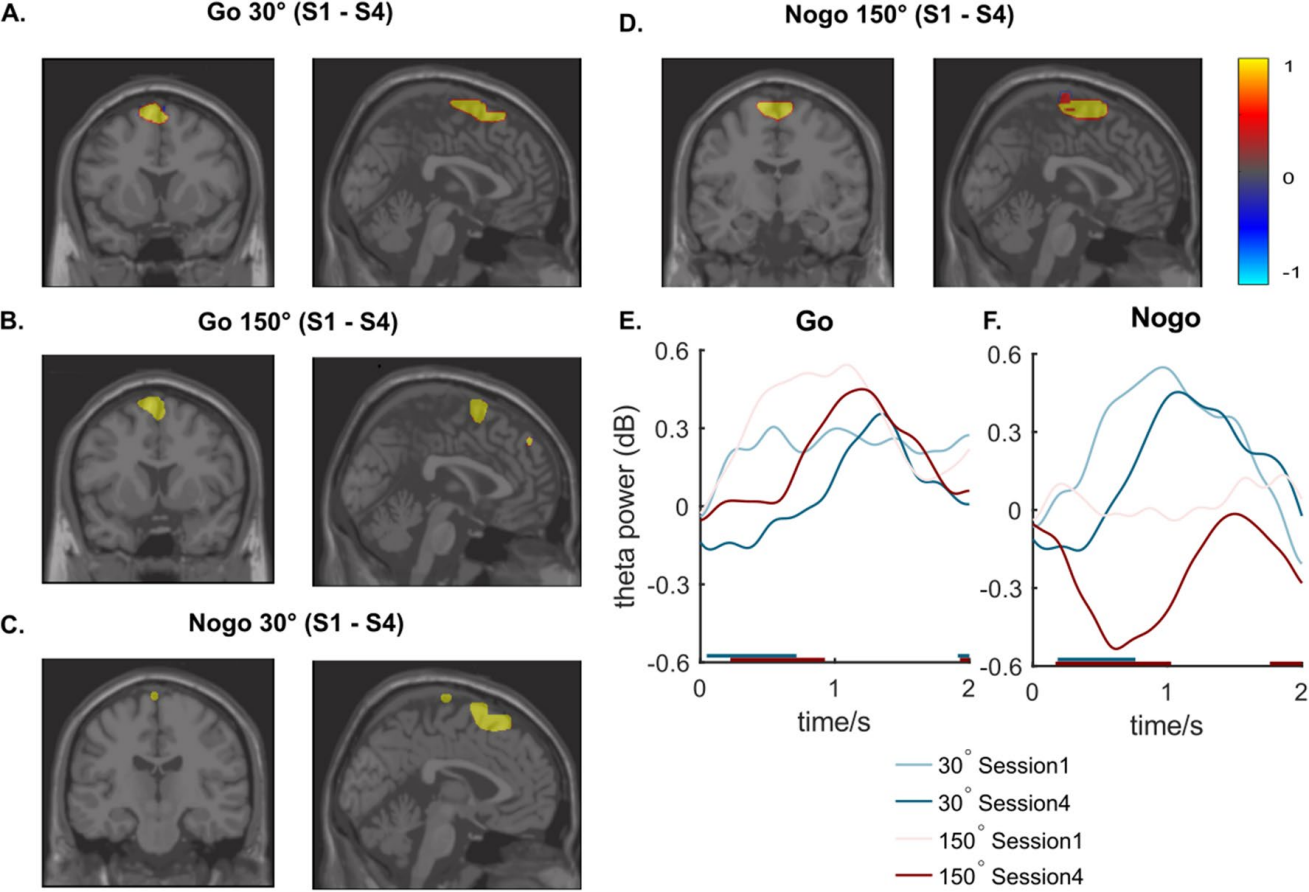
Anatomical regions of sources and task-evoked theta activity in source level. (**A**–**D**) present the anatomical regions showing a significant difference of task-related TBA between session S1 and session S4 in each condition. Figure parts (**E**) and (**F**) show the average baseline-normalized theta (4–7 Hz) activities (in dB) at the source level in the time window between 0 and 2 s after stimulus presentation. The bold lines parallel to x-axis indicate the time when the significant difference between S1 and S4 is observed. Red and blue colors represent the 30° and the 150° conditions, respectively.

Specifically, in the Go 30° condition (Fig. 4A) the difference between Session 1 and 4 was observed mostly in the supplementary motor area (BA6) and left dorsolateral superior frontal gyrus (BA8, 9). Other brain areas such as the paracentral lobule and precentral gyrus (BA4), and medial superior frontal gyrus (BA9) also revealed strong activity difference. In the Go 150° condition (Fig. 4B), the activity differences were also observed mostly in the supplementary motor area (BA6) and medial and dorsolateral superior frontal gyrus (BA8). Different from other conditions, significant difference in Go 150° was also observed in parietal, temporal and occipital lobes and cerebellum. In the Nogo conditions (Fig. 4C, D), the difference between Session 1 and session 4 was particularly shown in the supplementary motor area (BA6) and superior and medial frontal gyrus (BA8, 9). The difference was also found in the paracentral lobule and the precentral gyrus (BA4) for Nogo 30° and in the paracentral lobule (BA4) for Nogo 150°. Evidently the significant difference in frontal lobe is common in all conditions. The TBA (in dB) at the source level of frontal lobe is shown in Fig. 4E, F. In all conditions, a decrease was observed in the first second after stimulus presentation. Specifically, the significant change from S1 to S4 took place between 0.05 ~ 0.71 s for Go 30° condition (t(26) = 2.88, *p* = 0.014) and between 0.22 and 0.93 s for the Go 150° condition (t(26) = 3.47, *p* = 0.009). For the Nogo 30° condition, it was between 0.18 to 0.76 s (t(26) = 2.57, *p* = 0.022), and for the Nogo 150° condition, it was 0.17–1.03 s (t(26) = 3.09, *p* = 0.009).

### Correlations of source-level theta activity and pupil diameter data

**Figure 5:**
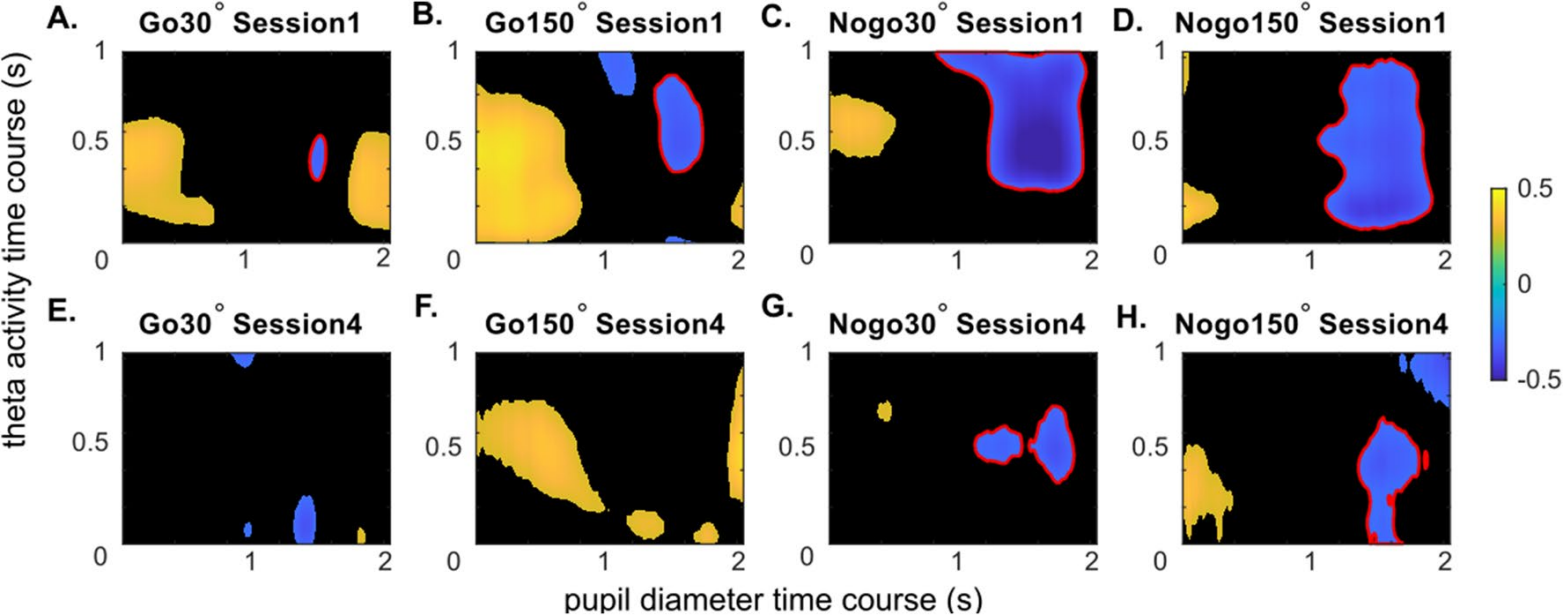
Correlation between theta activity and pupil diameter. Figure parts (**A**–**H**) shows the correlation coefficient matrix between source level task-related theta activities (dB) and phasic pupil diameter (baseline-corrected) in each condition and session. The x-axis refers to the time window of pupil diameter, which is 0 to 2 s; the y-axis refers to the time window of theta activity, which is 0 to 1 s. Only significant coefficients (*p* ≤ . 05) are shown. Black areas represent non-significant coefficients (*p* > .05). Red lines mark the boundaries of the negative correlation clusters of interest in each plot. The correlation coefficient (r) is colour coded. Warm colours denote positive correlations coefficients, cold colours negative correlation coefficients.

The correlation between pupil diameter and theta power is shown in Fig. 5.

**Figure 6:**
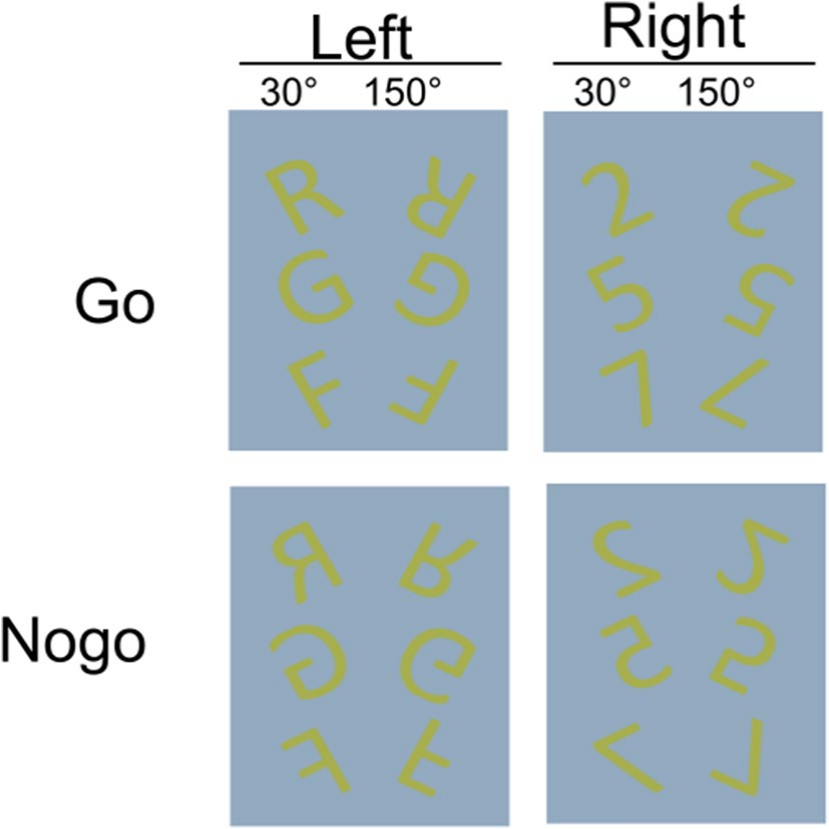
Stimulus categorization and corresponding responses. The colors of stimulus and the background are identical to the experiment's colours. Each panel represents all possible stimulus with a specific response. All stimuli for Go and Nogo trials are shown.

As indicated by the beamforming analysis, significant decreases of superior frontal TBA between session S1 and session S4 were mainly observed between 0 to 1 s. Therefore, correlations using theta activity in this time window are meaningful. Crucially, the pupil dilation dynamics lags behind for several hundred milliseconds^46,61^ and the pupil diameter reached the amplitude around 1 to 2 s (refer to Fig. 3A, B). Therefore, the correlation analysis results can only be interpreted in specific time windows and not the entire time range showing possible correlations can meaningfully be interpreted^27^. The relevant time window to interpret refers to a time window within the first second after stimulus presentation for superior frontal TBA (y-axis in Fig. 6) and between 1 and 2 s for the pupil diameter data (x-axis in Fig. 6). For that time window, strong negative correlations between the pupil diameter and superior frontal TBA were found in most conditions (*p* < 0.05). Importantly, the extent of strong negative correlations varied in different conditions and changed across sessions; that is correlations between the pupil diameter and superior frontal TBA were evident over longer time periods in session S1, compared to session S4. This is corroborated by a statistical analysis. For that, we extracted the negative correlation clusters centralized in the relevant time window for each condition and session. For comparison, we set the size of the largest cluster among all conditions to a value of 1 (session S1, Nogo 150° condition). Comparing the size of these clusters between session S1 (Fig. 5A–D) and in S4 (Fig. 5E–H), there was a noticeable decrease in the size in all conditions. In session S1, these negative correlation clusters were mostly located around 0.5 s of source level theta activity and between 1 and 2 s of pupil diameter, which suggested that stronger theta activity in source level around 0.5 s relates to lower pupil diameter after 1 s. Interestingly, in the conditions of larger rotation angel (150°), significant negative correlations were evident over longer time periods. Similarly, the duration/extend of negative correlations was also longer/larger in Nogo than in Go conditions (Go 30°: size = 0.04, r = −0.64, *p* = 0.036, R^2^ = −0.41; Go 150°: size = 0.25, r = −0.65, *p* = 0.03, R^2^ = −0.43; Nogo 30°: size = 0.97, r = −0.75, *p* = 0.009, R^2^ = −0.56; Nogo 150°: size = 1, r = −0.67, *p* = 0.021, R^2^ = −0.45). In session S4, the extent of negative correlations between pupil diameter and theta activity declined strongly. In the Nogo conditions, the negative correlation decreased more than half, but remained evident (30°: size = 0.25, r = −0.65, *p* = 0.031, R^2^ = −0.42; 150°: size = 0.33, r = −0.64, *p* = 0.034, R^2^ = −0.41). While in other conditions, the correlations were barely observed in S4, which suggested that the interrelation between theta activity and pupil diameter may extinguish with time.

## Discussion

The current study aimed to investigate time-on-task effects as conceptualized in the account by Kurzban et al.^5^ combining an analysis of theta band activity of pupil diameter data. For this purpose, we employed a combined Go/Nogo mental rotation task in an experiment lasting about 2.5 h. We interrelated phasic pupil dilation data as an indirect measure of norepinephrine system activity and engagement in a task with cognitive control-related source-level TBA derived from EEG beamforming.

The behavioral data showed a general increase in Go trials' reaction times and decrease in accuracy for both Go and Nogo trials. Despite the learning effect indicated by the decrease of reaction time and the increase of accuracies from the first session to the second session in most conditions, the general decline from the first/second session in reaction time and response accuracies are in line with previous studies^62–64^, indicating that this experiment successfully triggered time-on-task effects that resemble cognitive fatigue. The modulatory effect of working memory load was evident in behavioral performance: The rates of correct responses in the 150° condition were consistently lower than in 30° condition, and also the reaction time was longer. This finding is intuitive that a larger rotation angle increased the task difficulty^20^ thus impaired the performance. Declines in inhibitory control performance with time on task have also been shown previously^65^. However, most important in the current study is the modulation of brain-structure specific TBA and its relation to pupil diameter dynamics.

The pupil diameter is a viable metric to track mental effort^41–43^ or engagement in the task^44^. Changes in the phasic pupil diameter are evoked by task demands^45–49^. Likely changes in phasic pupil diameter reflect phasic norepinephrine responses^45–48^ for which the adaptive gain theory states that this is important to support demanding task-related decision or response selection processes^56,57^. In line with that, the phasic pupil dilation was consistently larger in 150° condition compared to the 30° condition. This is also in line with a previous study using similar paradigm^66^ and is well reasonable considering that larger rotation angles induce higher working memory load^67^. The change on pupil diameter also varied with rotation angles. In the 150° condition, the phasic pupil diameter revealed a particularly strong reduction, suggesting a modulatory role of cognitive demand in the effect of fatigue. Stronger phasic NE activity has also been associated with more task engagement^42,44,52–55^. The decrease of phasic pupil dilation from session S1 to S4 indicates that the efforts invested in task-related decision processes become smaller with time on task. However, the increase of baseline pupil diameter (refer supplemental material) suggests that NE system activity gradually turned into a tonic mode associated with disengagement from the current task^56^. Therefore, pupil diameter data suggest that the NE system's task-related modulatory processes and task engagement decrease. This is also predicted in the model by Kurzban et al.^5^, which states that the costs of continuing the task are represented and increase with time on task. This experience leads to disengagement in the primary task at hand and thereby reduces performance in the task. The modulations observed for the pupil diameter data can well explain the effects observed at the behavioral level. Nevertheless, the dynamic observed for the pupil diameter data has to be considered regarding the modulations in TBA.

For task-evoked TBA, there was a decrease in power from session S1 to S4 in Go and Nogo trials. Theta activity subserves cognitive control^58,68^ and response inhibition^27,28,32,69^. Accordingly, the decrease in task-related TBA (especially in Nogo trials) implies that cognitive control processes were diminished in session S4, compared to session S1, which fits the behavioral data. According to Kurzban et al.^5^, increasing time-on-task effect induces task disengagement. The pupil diameter data also suggest the latter. Notably, the beamforming analysis of TBA shows that these fatigue-related effects on cognitive control processes occur in the superior frontal cortex. TBA in the superior frontal cortex has frequently been shown to related to response inhibition processes and a cortical response inhibition network^27,30,36^ and evidence also shows that these regions play a role in working memory processes^37–40^. However, the activity modulations in the superior frontal gyrus were also evident in the medial portions of this gyrus. Medial frontal TBA, including superior frontal regions^58,70^ has been suggested to reflect a realization of the need for cognitive control related to novel information, errors and conflicts^58,70^, which matches the features of Nogo stimuli. With time-on task, TBA-related cognitive control processes in superior frontal regions including their medial portion decline. This fits well to the account's assumptions by Kurzban et al.^5^ according to which particularly prefrontal cortical regions show time-on-task effects^4,5^, suggesting the decreased working memory processes, and the increased disengagement of novelty detection in Nogo tasks. Of note, superior frontal TBA and pupil diameter dynamics were directly related across participants, and this correlation shows time-on-task-dependent modulations: In session S1, there was a negative correlation between phasic pupil diameter and task-evoked superior frontal TBA in all conditions. That is, a smaller phasic pupil diameter was related to higher task-evoked superior frontal TBA. Several conceptions on the precise role of TBA in cognitive control suggest that medial frontal TBA suggest the need to engage in cognitive control^58,59^. From that perspective, the negative correlation between phasic pupil diameter and superior frontal TBA seems reasonable as especially a smaller phasic pupil diameter may reflect lower efforts/engagement invested in the task. When there is a lower effort invested, cognitive control processes become particularly demanding. Vice versa, when there is strong superior frontal TBA, it is not necessary to invest high efforts in the task. Due to the correlative nature of the data, it cannot be decided whether the degree of superior frontal TBA (i.e., allocated cognitive control) drives the pupil diameter effect (i.e. effort invested in the task), or whether the pupil diameter (i.e. effort invested in the task) determines the superior frontal TBA (i.e. cognitive control being allocated). However, the first scenario is more like for the following reason. Superior frontal TBA was maximal ~ 500 ms after stimulus presentation (cf. Figure 4). Crucially, the pupil dilation dynamics lags behind relevant neuronal activity for several hundred milliseconds^46,61^ and the negative correlations with superior frontal TBA were evident between 1 and 2 s after stimulus presentation for the pupil diameter data. This temporal shift makes it likely that superior frontal TBA ‘drives’ pupil diameter dynamics. Therefore, the data suggest that a higher degree of cognitive control allocated to the task leads to smaller task engagement. Notably, in session S4, pupil diameter and superior frontal TBA correlation were still evident, but only across much shorter time periods. This is directly evidenced by comparing the size of negative correlation clusters between pupil diameter and superior frontal TBA (cf. Figure 5) between session S1 and S4. In session S4, the size of the negative correlation cluster was always smaller than in session S1. This suggests that with time on task, the inter-relation by superior frontal TBA and pupil diameter modulations vanishes. While there was a strong relationship between cognitive control and task-related effort at the beginning of the task, the two aspects seem to be relatively unrelated towards the end of the task. The extent of cognitive control and the extent of invested effort thus seem to become independent of each other with increasing time-on-task effects. The theoretical account by Kurzban et al.^5^ proposes that with the expected benefit to engage in a task and to allocate full processing resources to the task declines. The current data suggest that it is a decoupling of the amount of prefrontal cognitive control resources useable for a task and the amount of effort invested in the task that characterizes time-on-task effects.

In summary, we examined time-on-task effects in cognitive control on a neurophysiological level using a working-memory modulated response inhibition task and inter-relate prefrontal neuroanatomical region-specific theta-band activity (TBA) with pupil diameter data using EEG-beamforming approaches. We showed task performance declines with time-on tasks, which was paralleled by a concomitant decreases of task-evoked superior frontal gyrus TBA and a reduction in phasic pupil diameter modulations. Correlating the time course superior frontal TBA with the time course of the pupil diameter data, it is shown that there was a strong relation between task-evoked superior frontal TBA indexing cognitive control and effort/task engagement indexed by phasic pupil diameter modulations at the beginning of the experiment. Importantly, this inter-relation vanished at the end of the experiment. The conceptual advance is that the data suggest for a decoupling of the amount of prefrontal cognitive control resources useable for a task and the amount of effort invested in the task that characterizes time-on-task effects for prefrontal cortex functions. Despite the findings about time-on-task effects in cognitive control, there was also a learning effect in the behavioural data. However, in the design it is not possible to isolate concomitant learning and fatigability processes. Further studies are needed to separately examine these two effects.

## Materials and methods

### Participants

*N* = 35 healthy volunteers from age 18 to 40 (16 males; mean age 26.4; 34 right-handed) were enrolled in the experiment. Participants reported no history of neurological or mental illness within the last six months. All participants had a normal or corrected-to-normal vision. Participants were instructed to not to drink caffeinated beverages before the experiment. Participants were informed about time duration and project mission before the experiment. They were reimbursed for full participation (€ 35). For the data analysis, subjects were only included if behavioral data, pupil diameter data and EEG data were available for all four sessions. Participants who did not perform the task for the entire duration or participants whose behavioral performance was below the chance level in one of the sessions were excluded from data analysis. The final sample for data analysis consisted of *N* = 27 subjects. All participants provided written informed consent before any study procedure was applied. This study was approved by the Ethics committee of Medical faculty of the TU Dresden and was conducted in accordance with the Declaration of Helsinki.

We collected data on the Fatigue Scale of Motor and Cognitive Functions (FSMC), where cognitive or motor score lower than 22 shows no cognitive or motor fatigue while a cognitive or motor score between 22 and 28 shows mild fatigue^71^. Similarly, we examined the Beck Depression Inventory (BDI) where scores lower than 10 indicate that there are no depressive symptoms^60^.

### Procedures

To avoid possible daytime effects, all experiments started around 9:00 AM. Participants were seated in a dimly lit room at a distance of 50–70 cm in front of a 24 inches display. To reduce the effect of luminance on pupil diameter and eliminate pupil dilation caused by irrelevant stimulation from brightness, nearly equiluminant colors were deliberately assigned for stimuli (RGB code 168, 175, 77) and background (RGB code 147, 170, 192) as shown in Fig. 6.

An eye tracker was placed under the display with an adjustable degree of 20° to 40° at the horizontal plane. Participants were required to keep the sitting position as stable as possible to avoid losing eye tracks. To measure participants’ general fatigue levels and psychological well-being at the day of testing, the Fatigue Scale for Motor and Cognitive Functions (FSMC)^71^ and Beck’s Depression Inventory (BDI)^60^ were administered before the experiment. After participants finished these questionnaires, EEG caps were prepared. Subsequently, the experimenter explained the task and experimental requirements and guided participants to practice the task for at least 24 trials until they understood the task and requirements. Before the formal experiment, participants were instructed to calibrate the eye tracker. The formal experiment included 4,800 trials in total, and each trial took about 2 s.

To collect the pupil diameter baseline data, several fixation periods were assigned during the experiment. During fixation sessions, no trials were presented. Instead, only a fixation cross was displayed in the center of the screen. At the start and the end of the experiment, the fixation periods lasted for two minutes. During the experiment, considering that a long fixation period without any task may strongly counteract the emergence of fatigue and time on task effects^2^, the fixation periods lasted only ten seconds because a previous study by Lim et al.^72^ showed that long-term effect of time-on-task could persist despite short breaks of 12 to 28 s. The number of short fixation periods was 19, and they were evenly distributed during the experiment. The overall experiment took about 2 h and 40 min. Importantly, no break was included during the entire experiment, since a break lasting for several minutes can restore performance in cognitive control tasks^2^. Before the experiment, participants were informed about their rights to stop the experiment at any time without reason.

### Task and stimulus

We used an established working memory modulated Go/Nogo task^19,20^, a combined Go/Nogo mental rotation task. The paradigm used was adapted from a previous study by our group^19,20^. In each trial, a fixation cross was first presented in the screen for a random time duration of 900 to 1300 ms. Then, a stimulus was displayed for 1100 ms. Participants were required to respond only to Go trials in the duration of stimulus presentation by pressing buttons. During Nogo trials, participants were asked to refrain from pressing a button. Non-mirrored stimuli were Go trials. The left “Ctrl” key should be pressed if the stimulus was a letter; the right “Ctrl” key had to be pressed if the stimulus was a number. Nogo trials were indicated by mirrored letters or numbers. To avoid ambiguity of mirrored and non-mirrored letters and numbers, asymmetric numbers 2, 5 and 7 and letters F, G, R were employed as stimuli. We used letter and number stimuli because these do not evoke sex-dependent differences in the mental rotation process^73^. In Go and Nogo trials, each stimulus was either rotated 30° or 150° clockwise. This was the case for mirrored and non-mirrored images. This rotation angle manipulation was included to vary working memory load^67,74,75^. A summary of target stimuli and corresponding responses under each condition is illustrated in Fig. 6. To increase response tendency in the participants, Go and Nogo stimuli were presented in a ratio of 7:3^19^. The order of stimuli was randomized to avoid that participants can predict upcoming trials. The order of stimuli was the same for all participants. To analyze time-on-task effects, behavioral data from each participant were divided into four sessions. Each session consisted of 1200 trials for complete behavioural data, including 840 Go trials and 360 Nogo trials. In each session of Go trials and Nogo trials, the number of trials under conditions of 30°-rotated and 150°-rotated were equal. Only trials with correct responses in each session, namely, hit and correct rejection, were selected for further analysis. Trials with correct responses in each session were categorized to 4 conditions: 30°-rotated Go (Go 30°), 150°-rotated Go (Go 150°), 30°-rotated Nogo (Nogo 30°) and 150°-rotated Nogo (Nogo 150°). The same approach was also applied to EEG data after pre-processing and pupil diameter data after synchronization and pre-processing.

### EEG recording and analysis

EEG data were recorded from 60 Ag/AgCl electrodes arranged in equidistant positions. EEG data pre-processing was performed using the BrainVision Recorder software package (Brain Products, Inc.). The ground and reference electrodes were positioned at coordinates theta = 58, phi = 78 and theta = 90, phi = 90, respectively. The recording sampling rate was 500 Hz. The raw EEG data were pre-processed with Brain Vision Analyzer 2 software package (Brain Products Inc). First, the initial sampling rate was reduced to 256 Hz. The data were then filtered by Infinite Impulse Response (IIR) filters with high-pass of 0.5 Hz and low-pass of 40 Hz at a slope of 48 db/oct each. Additionally, a notch filter of 50 Hz was applied. Channels with no activity and defective channels with high noise were deleted after filtering. After that, a new average reference was calculated. Irregular, technical artefacts were removed through a manual inspection. An independent component analysis (ICA, infomax algorithm) was then applied to remove regular artefacts such as eye blinks, eye movements, and pulses (mean discarded ICs = 6.2 ± 2.4). A topographic interpolation was then applied to reconstruct the previously discarded electrode channels. Because we focus on low frequency activities in this study, an additional low-pass filter of 20 Hz was applied. After pre-processing, the continuous EEG data were segmented to 4 sessions. Within these four sessions, trials were defined and categorized using the same method as in behavioral data (see *Task and stimulus*). For each trial, the time of stimulus presentation was used as a locking point. To avoid cone of influence effects that may occur during time–frequency decomposition, each segment's length was set to 5000 ms, starting from 2000 ms before the locking time point and ended at 3000 ms after stimulus presentation (locking time point). Then, an automated artefact rejection procedure was applied to remove residual artefacts in the segmented data. The criteria followed the default settings in Brain Vision Analyzer 2, set between 200 ms before the event and 200 ms after the event. The maximum allowed voltage step was 30 µV/ms; the maximum difference of values in intervals was 100 µV with an interval length of 200 ms. Minimum and maximum allowed amplitude were −150 µV and 150 µV respectively, and the lowest allowed activities were 0.5 µV in an interval of 100 ms. Thereafter, a baseline correction from −200 to 0 ms before stimulus onset was applied.

For the time–frequency decomposition, a current-source-density (CSD) transformation^76^ was applied. After that, the time–frequency analysis was run using Morlet wavelets (*w*) in the time domain (*t*) to different frequencies (*f*). Total wavelet powers were calculated as in Eq. 1.

1 $$w\left(t,f\right)=A\mathrm{exp}(\frac{-{t}^{2}}{2{\sigma }_{t}^{2}})\mathrm{exp}\left(2i\pi ft\right)$$

where *t* is the time, $A={\left({\sigma }_{t}\sqrt{\pi }\right)}^{-1/2}$, ${\sigma }_{t}$ is the wavelet duration. The time–frequency decomposition was computed on single trials data for all segmented conditions and then averaged for each participant. Further, a baseline correction was conducted using data in the time interval from −200 to 0 ms. We used decibel (dB) conversion, which is a log-transform on the strength of signal relative to the baseline. To examine theta-band activity, we focused on the frequency range between 4 and 7 Hz. To identify electrode sites showing significant theta-band activity, cluster-based permutation tests were computed in Fieldtrip. As our study focuses on the change of theta activities across time, we compared the time–frequency results between session S1 and S4 for each condition of Go/Nogo and 30°/150°. We applied the Monte-Carlo method to obtain the reference distributions for cluster-based permutation tests using 500 random draws. The cluster-based permutation tests (*p* < 0.05) were run based on dependent t-test results for every electrode within the first second of the averaged theta activities.

### Source estimation methods

The beamforming analysis was conducted according to the procedure used in previous studies^27,77^ using processed EEG data without CSD-transformation. For the beamforming analysis and integration (correlation) of the beamforming analysis with the analysis of the pupil diameter data, we focused on the first session (S1) and the fourth session (S4). This was a data-driven decision since the behavioral data revealed the most substantial performance effects between these blocks. For the beamforming analysis, session S1 was contrasted with session S4. Two beamformers were computed successively: (i) a dynamic imaging of coherent sources beamformer (DICS)^78^ and (ii) a linear constraint minimum variance beamformer (LCMV)^79^ was applied on the DICS-beamformed data. This last LCMV beamforming step is necessary since the DICS beamformer only yields the location of activity in the brain, but not the time course in this sources. To derive the time course of activity in the DICS-reconstructed beamforming source, it is necessary to employ an LCMV beamformer^27^. It is the time course of TBA activity that is correlated with the pupil diameter data (see "statistical analysis" below), which is not possible without LCMV beamforming after DICS beamforming^27^.

The DICS beamformer was computed in single-subject level for each of the four conditions (Go 30°, Go 150°, Nogo 30° and Nogo 150°) separately. To identify the neuroanatomical regions that reflect theta activity changes from the first session to the last session, the frequency of interest was set to 4 ~ 7 Hz, spanning the entire frequency band without the edge frequency adjacent to the delta and alpha frequency band. First, we adopted a head model developed by Nolte^80^ to construct a forward model and perform beamforming. Then, the EEG electrodes were realigned to the forward model, and a leadfield matrix was prepared by discretizing forward model’s brain volume to a regular 3-D grid of 1 cm resolution. Considering that the pre-stimulus theta activity may vary with time on task, we constructed DICS beamformer based on both pre- and post-stimulus data. The time window of pre-stimulus data was set to −750 to 0 ms according to the stimulus onset, and for post-stimulus data, it was 250 to 1000 ms based on the observation of time frequency decomposition results after cluster-based permutation (see "Results"). Theta activity was assessed for both segments through a spectral analysis where a multitaper frequency transformation was implemented to generate the power and spectral density matrix. A spatial filter was computed based on frequency transformation results of both pre- and post-stimulus data from all chosen sessions with regularization parameter as 5%. This filter was then applied to each session to extract the source and its power for the pre- and post-stimulus segment. For each session, the beamformer was then constructed with post-stimulus data baseline-normalized by pre-stimulus data. Beamformer contrasts were then computed as normalized power difference between session S1 and session S4 (Eq. 2) and were used to interpolate source.

2 $${P}_{diff}=\frac{\frac{{P}_{S{4}_{post}}}{{P}_{S{4}_{pre}}}-\frac{{P}_{S{1}_{post}}}{{P}_{{S4}_{pre}}}}{\frac{{P}_{S{4}_{post}}}{{P}_{S{4}_{pre}}}+\frac{{P}_{S{1}_{post}}}{{P}_{S{4}_{pre}}}}$$

Beamforming using Fieldtrip relies on a contrast between 2 conditions. It is not possible to model more than 2 conditions in the calculation of the beamforming sources using Fieldtrip (this differs from fMRI approaches where full factorial models can be calculated using SPM and where multiple levels of a factor can be modeled). Therefore, one has to choose 2 conditions when running beamforming analyses. This is the reason, why, opposed to the task performance behavioral data, only the condition S1 and S4 were compared. From a theoretical point of view, the fatigability effect is maximal between S1 and S4. Therefore, we chose this approach. Moreover, since all conditions cannot be modelled at once in Fieldtrip one runs into multiple comparison problems when contrasting all conditions against each other. Only voxels revealing activity in the top 1% of all voxels were located anatomically to have a conservative estimate of only the most active voxels. The anatomical regions covered in each of these clusters were mapped on the Fieldtrip head model template “standard_mri”. The original construction of this head model is detailed by Holmes et al.^81^. Finally, these clusters and their corresponding anatomical regions were used in the following LCMV beamforming steps.

The LCMV beamforming was implemented to reconstruct the activities in the region of interest (ROI) as identified in the DICS beamforming step. For the LCMV beamformer, on the purpose of comparison among conditions, only the coordinates of common anatomical regions in most conditions (i.e. regions in the frontal lobe, see "Results") were grouped as source position indices, and a covariance matrix was computed through averaging all single trials in each participant for each condition. The source indices were later applied to previously pre-processed EEG single-trial data to generate an adaptive spatial filter for subsequent data reconstruction for S1 and S4. In every single trial, the source-reconstructed data were then averaged in the dimension of source indexes to generate source level time series. The source level time series was then time–frequency decomposed using Morlet wavelets, and theta oscillations (4 ~ 7 Hz) were isolated and averaged across frequencies at the single-subject level. A baseline correction was computed from a time interval of −200 to 0 ms and was applied to the averaged theta oscillation, which yielded the theta activity in source level for session S1 and S4 in the Go 30°, Go 150°, Nogo 30° and Nogo 150° conditions, separately.

### Pupil diameter recordings and processing

Pupil diameter recordings and analyses were conducted and aligned with the EEG data using previously established protocols^20,27,66^. The pupil diameter data were recorded using a RED 500 eye tracking device and the software iView X (SensoMotoric Instruments GmbH) with a 256 Hz sampling rate. Right before the experiment, the eye tracker was calibrated by a 9-point calibration method. During the experiment, eye movements were simultaneously recorded with EEG data. The eye-tracking recording software automatically interpolated eye blinks. The pupil diameter data and raw EEG data from same subjects were synchronized with the EYE-EEG extension^82^ for EEGLab (http://www2.hu-berlin.de/eyetracking-eeg/)83 in MATLAB 2019a (Mathworks, Inc.) according to the identical start and end markers in both data files. The synchronization aligned the pupil diameter data with all markers in EEG data. For data analysis and integration of the pupil diameter data with the EEG data, a low pass filter was applied (IIR filter with 20 Hz at a 48 dB/oct slope). Segmentation and baseline correction was also conducted in the same way as the EEG data.

### Statistical analysis

The statistical analysis of the behavioral data and pupil diameter data was conducted for each of 4 conditions (Go 30°, Go 150°, Nogo 30°, Nogo 150°) in each of 4 sessions (S1 to S4). For the behavioral data, the rate of correct responses and mean reaction times (RTs) in Go trials and the correct rejection rate in Nogo trials, were used. For pupil diameter data, pupil diameter peak amplitude was defined as the maximum value of local peaks after stimulus presentation. Accordingly, the peak latency was then defined as the time point of the peak. All parameters were analyzed using repeated measures ANOVAs. The data were analyzed for Go and Nogo conditions separately using the within-subject factors ‘session’ (S1, S2, S3, S4) and ‘rotation angle’ (30°, 150°). The Greenhouse–Geisser correction was applied, and post-hoc tests were Bonferroni-corrected when necessary.

Correlation of pupil diameter and the LCMV-beamformed EEG data were calculated in the S1 and S4 sessions for the Go 30°, Go 150°, Nogo 30° and Nogo 150° conditions, separately. We calculated Pearson correlations between the pupil diameter data and LCMV-reconstructed time course of theta activity in the identified sources for each condition and session. For the correlation of the LCMV-reconstructed source activity data with the pupil diameter data, we only used source-level theta activities in a time bin of 0 ~ 1 s after stimulus presentation, because significant differences between S1 and S4 sessions were observed in this period (see "Results"). For that, we separately averaged pupil diameter data on the single-subject level. Due to the low signal-to-noise ratio of single-trial EEG data, no within-subject correlations between pupil diameter data and EEG data were calculated on a trial-by-trial manner^66^. Finally, every theta activity data time bin was correlated with every pupil diameter data time bin across all subjects.

## Supplementary Information

Supplementary Information.

## Data Availability

The datasets generated during and/or analysed for the current study are available at the Open Science Framework https://osf.io/afyux We used standard software packages as described in the methods section. Non-standard Matlab scripts can be retrieved from the Open Science Framework https://osf.io/afyux.

## References

[1] Lal SKL, Craig A (2001). A critical review of the psychophysiology of driver fatigue. Biol. Psychol..

[2] Möckel T, Beste C, Wascher E (2015). The effects of time on task in response selection: an ERP study of mental fatigue. Sci. Rep..

[3] Wascher E (2014). Frontal theta activity reflects distinct aspects of mental fatigue. Biol. Psychol..

[4] Wang C, Trongnetrpunya A, Samuel IBH, Ding M, Kluger BM (2016). Compensatory neural activity in response to cognitive fatigue. J. Neurosci..

[5] Kurzban R, Duckworth A, Kable JW, Myers J (2013). An opportunity cost model of subjective effort and task performance. Behav. Brain Sci..

[6] Baumeister RF, Bratslavsky E, Muraven M, Tice DM (1998). Ego depletion: is the active self a limited resource?. J. Pers. Soc. Psychol..

[7] Nielsen B, Hyldig T, Bidstrup F, González-Alonso J, Christoffersen GRJ (2001). Brain activity and fatigue during prolonged exercise in the heat. Pflüg. Arch..

[8] Webster DM, Richter L, Kruglanski AW (1996). On leaping to conclusions when feeling tired: mental fatigue effects on impressional primacy. J. Exp. Soc. Psychol..

[9] Wharton GK (1938). The fatigue syndrome. Can. Med. Assoc. J..

[10] Boksem MAS, Tops M (2008). Mental fatigue: costs and benefits. Brain Res. Rev..

[11] Hockey, G. R. J. A motivational control theory of cognitive fatigue. in *Cognitive fatigue: Multidisciplinary perspectives on current research and future applications* 167–187 (American Psychological Association, 2011). 10.1037/12343-008.

[12] Robinson M (2010). Plasma IL-6, its soluble receptors and F2-isoprostanes at rest and during exercise in chronic fatigue syndrome. Scand. J. Med. Sci. Sports.

[13] Diamond A (2013). Executive functions. Annu. Rev. Psychol..

[14] Lee TG, D’Esposito M (2012). The dynamic nature of top-down signals originating from prefrontal cortex: a combined fMRI–TMS study. J. Neurosci..

[15] Zanto TP, Rubens MT, Thangavel A, Gazzaley A (2011). Causal role of the prefrontal cortex in top-down modulation of visual processing and working memory. Nat. Neurosci..

[16] Lehto JE, Juujärvi P, Kooistra L, Pulkkinen L (2010). Dimensions of executive functioning: Evidence from children. Br. J. Dev. Psychol..

[17] Miyake A (2000). The unity and diversity of executive functions and their contributions to complex “frontal lobe” tasks: a latent variable analysis. Cognit. Psychol..

[18] Barch DM (1997). Dissociating working memory from task difficulty in human prefrontal cortex. Neuropsychologia.

[19] Chmielewski WX, Mückschel M, Stock A-K, Beste C (2015). The impact of mental workload on inhibitory control subprocesses. Neuroimage.

[20] Chmielewski WX, Mückschel M, Ziemssen T, Beste C (2017). The norepinephrine system affects specific neurophysiological subprocesses in the modulation of inhibitory control by working memory demands. Hum. Brain Mapp..

[21] Cohen JD (1997). Temporal dynamics of brain activation during a working memory task. Nature.

[22] Curtis CE, D’Esposito M (2003). Persistent activity in the prefrontal cortex during working memory. Trends Cogn. Sci..

[23] D’Esposito M, Postle BR, Rypma B (2000). Prefrontal cortical contributions to working memory: evidence from event-related fMRI studies. Exp. Brain Res..

[24] Chmielewski WX, Mückschel M, Dippel G, Beste C (2016). Concurrent information affects response inhibition processes via the modulation of theta oscillations in cognitive control networks. Brain Struct. Funct..

[25] De Blasio FM, Barry RJ (2013). Prestimulus delta and theta determinants of ERP responses in the Go/NoGo task. Int. J. Psychophysiol. Off. J. Int. Organ. Psychophysiol..

[26] Dippel G, Chmielewski W, Mückschel M, Beste C (2016). Response mode-dependent differences in neurofunctional networks during response inhibition: an EEG-beamforming study. Brain Struct. Funct..

[27] Dippel G, Mückschel M, Ziemssen T, Beste C (2017). Demands on response inhibition processes determine modulations of theta band activity in superior frontal areas and correlations with pupillometry: implications for the norepinephrine system during inhibitory control. Neuroimage.

[28] Huster RJ, Enriquez-Geppert S, Lavallee CF, Falkenstein M, Herrmann CS (2013). Electroencephalography of response inhibition tasks: functional networks and cognitive contributions. Int. J. Psychophysiol. Off. J. Int. Organ. Psychophysiol..

[29] Isabella S, Ferrari P, Jobst C, Cheyne JA, Cheyne D (2015). Complementary roles of cortical oscillations in automatic and controlled processing during rapid serial tasks. Neuroimage.

[30] Mückschel M, Dippel G, Beste C (2017). Distinguishing stimulus and response codes in theta oscillations in prefrontal areas during inhibitory control of automated responses. Hum. Brain Mapp..

[31] Pscherer C, Mückschel M, Summerer L, Bluschke A, Beste C (2019). On the relevance of EEG resting theta activity for the neurophysiological dynamics underlying motor inhibitory control. Hum. Brain Mapp..

[32] Quetscher C (2015). Striatal GABA-MRS predicts response inhibition performance and its cortical electrophysiological correlates. Brain Struct. Funct..

[33] Vahid A, Mückschel M, Neuhaus A, Stock A-K, Beste C (2018). Machine learning provides novel neurophysiological features that predict performance to inhibit automated responses. Sci. Rep..

[34] Hsieh L-T, Ranganath C (2014). Frontal midline theta oscillations during working memory maintenance and episodic encoding and retrieval. Neuroimage.

[35] Jensen O (2006). Maintenance of multiple working memory items by temporal segmentation. Neuroscience.

[36] Adelhöfer N, Mückschel M, Teufert B, Ziemssen T, Beste C (2019). Anodal tDCS affects neuromodulatory effects of the norepinephrine system on superior frontal theta activity during response inhibition. Brain Struct. Funct..

[37] Boisgueheneuc F (2006). Functions of the left superior frontal gyrus in humans: a lesion study. Brain.

[38] Owen AM (2000). The role of the lateral frontal cortex in mnemonic processing: the contribution of functional neuroimaging. Exp. Brain Res..

[39] Rypma B, Prabhakaran V, Desmond JE, Glover GH, Gabrieli JD (1999). Load-dependent roles of frontal brain regions in the maintenance of working memory. Neuroimage.

[40] Rypma B, D’Esposito M (1999). The roles of prefrontal brain regions in components of working memory: effects of memory load and individual differences. Proc. Natl. Acad. Sci. USA.

[41] Eckstein, M. K., Guerra-Carrillo, B., Miller Singley, A. T. & Bunge, S. A. Beyond eye gaze: What else can eyetracking reveal about cognition and cognitive development? *Dev. Cogn. Neurosci.* **25**, 69–91 (2017). 10.1016/j.dcn.2016.11.001 PMC6987826 27908561

[42] Hopstaken JF, van der Linden D, Bakker AB, Kompier MAJ (2015). The window of my eyes: Task disengagement and mental fatigue covary with pupil dynamics. Biol. Psychol..

[43] van der Wel P, van Steenbergen H (2018). Pupil dilation as an index of effort in cognitive control tasks: a review. Psychon. Bull. Rev..

[44] Gilzenrat MS, Nieuwenhuis S, Jepma M, Cohen JD (2010). Pupil diameter tracks changes in control state predicted by the adaptive gain theory of locus coeruleus function. Cogn. Affect. Behav. Neurosci..

[45] Gabay S, Pertzov Y, Henik A (2011). Orienting of attention, pupil size, and the norepinephrine system. Atten. Percept. Psychophys..

[46] Joshi S, Li Y, Kalwani RM, Gold JI (2016). Relationships between pupil diameter and neuronal activity in the locus coeruleus, colliculi, and cingulate cortex. Neuron.

[47] Reimer J (2016). Pupil fluctuations track rapid changes in adrenergic and cholinergic activity in cortex. Nat. Commun..

[48] Wolff N, Mückschel M, Ziemssen T, Beste C (2018). The role of phasic norepinephrine modulations during task switching: evidence for specific effects in parietal areas. Brain Struct. Funct..

[49] Zhao S (2019). Pupil-linked phasic arousal evoked by violation but not emergence of regularity within rapid sound sequences. Nat. Commun..

[50] Anderson CJ, Colombo J (2009). Larger tonic pupil size in young children with autism spectrum disorder. Dev. Psychobiol..

[51] Beatty J (1982). Phasic not tonic pupillary responses vary with auditory vigilance performance. Psychophysiology.

[52] Hong L, Walz JM, Sajda P (2014). Your eyes give you away: prestimulus changes in pupil diameter correlate with postst. PLoS ONE.

[53] Hou RH, Freeman C, Langley RW, Szabadi E, Bradshaw CM (2005). Does modafinil activate the locus coeruleus in man? Comparison of modafinil and clonidine on arousal and autonomic functions in human volunteers. Psychopharmacology.

[54] Jepma M, Nieuwenhuis S (2010). Pupil diameter predicts changes in the exploration–exploitation trade-off: evidence for the adaptive gain theory. J. Cogn. Neurosci..

[55] Murphy PR, Robertson IH, Balsters JH, O’connell RG (2011). Pupillometry and P3 index the locus coeruleus–noradrenergic arousal function in humans. Psychophysiology.

[56] Aston-Jones G, Cohen JD (2005). An integrative theory of locus coeruleus-norepinephrine function: adaptive gain and optimal performance. Annu. Rev. Neurosci..

[57] Nieuwenhuis S, Aston-Jones G, Cohen JD (2005). Decision making, the P3, and the locus coeruleus: norepinephrine system. Psychol. Bull..

[58] Cavanagh JF, Frank MJ (2014). Frontal theta as a mechanism for cognitive control. Trends Cogn. Sci..

[59] Womelsdorf T, Vinck M, Leung LS, Everling S (2010). Selective theta-synchronization of choice-relevant information subserves goal-directed behavior. Front. Hum. Neurosci..

[60] Beck AT, Steer RA, Ball R, Ranieri WF (1996). Comparison of beck depression inventories-IA and-II in psychiatric outpatients. J. Pers. Assess..

[61] Nieuwenhuis S, De Geus EJ, Aston-Jones G (2011). The anatomical and functional relationship between the P3 and autonomic components of the orienting response. Psychophysiology.

[62] Boksem MAS, Meijman TF, Lorist MM (2005). Effects of mental fatigue on attention: an ERP study. Cogn. Brain Res..

[63] Kato Y, Endo H, Kizuka T (2009). Mental fatigue and impaired response processes: event-related brain potentials in a Go/NoGo task. Int. J. Psychophysiol..

[64] Smit AS, Eling PATM, Coenen AML (2004). Mental effort causes vigilance decrease due to resource depletion. Acta Psychol. (Amst.).

[65] Guo Z (2018). The impairing effects of mental fatigue on response inhibition: an ERP study. PLoS ONE.

[66] Mückschel M, Chmielewski W, Ziemssen T, Beste C (2017). The norepinephrine system shows information-content specific properties during cognitive control: evidence from EEG and pupillary responses. Neuroimage.

[67] Band GPH, Kok A (2000). Age effects on response monitoring in a mental-rotation task. Biol. Psychol..

[68] Cavanagh JF, Shackman AJ (2015). Frontal midline theta reflects anxiety and cognitive control: Meta-analytic evidence. J. Physiol. Paris.

[69] Beste C (2017). Striosomal dysfunction affects behavioral adaptation but not impulsivity—evidence from X-linked dystonia-parkinsonism. Mov. Disord..

[70] Cohen MX (2014). A neural microcircuit for cognitive conflict detection and signaling. Trends Neurosci..

[71] Penner I (2009). The fatigue scale for motor and cognitive functions (FSMC): validation of a new instrument to assess multiple sclerosis-related fatigue. Mult. Scler. J..

[72] Lim J, Teng J, Wong KF, Chee MWL (2016). Modulating rest-break length induces differential recruitment of automatic and controlled attentional processes upon task reengagement. Neuroimage.

[73] Jansen-Osmann P, Heil M (2007). Suitable stimuli to obtain (no) gender differences in the speed of cognitive processes involved in mental rotation. Brain Cogn..

[74] Beste C, Heil M, Konrad C (2010). Individual differences in ERPs during mental rotation of characters: lateralization, and performance level. Brain Cogn..

[75] Heil M (2002). The functional significance of ERP effects during mental rotation. Psychophysiology.

[76] Nunez PL, Pilgreen KL (1991). The spline-laplacian in clinical neurophysiology: a method to improve EEG spatial resolution. J. Clin. Neurophysiol..

[77] Mückschel M, Stock A-K, Dippel G, Chmielewski W, Beste C (2016). Interacting sources of interference during sensorimotor integration processes. Neuroimage.

[78] Gross J (2001). Dynamic imaging of coherent sources: studying neural interactions in the human brain. Proc. Natl. Acad. Sci..

[79] Veen BDV, Drongelen WV, Yuchtman M, Suzuki A (1997). Localization of brain electrical activity via linearly constrained minimum variance spatial filtering. IEEE Trans. Biomed. Eng..

[80] Nolte G (2003). The magnetic lead field theorem in the quasi-static approximation and its use for magnetoencephalography forward calculation in realistic volume conductors. Phys. Med. Biol..

[81] Holmes CJ (1998). Enhancement of MR images using registration for signal averaging. J. Comput. Assist. Tomogr..

[82] Dimigen O, Sommer W, Hohlfeld A, Jacobs AM, Kliegl R (2011). Coregistration of eye movements and EEG in natural reading: analyses and review. J. Exp. Psychol. Gen..

[83] Delorme A, Makeig S (2004). EEGLAB: an open source toolbox for analysis of single-trial EEG dynamics including independent component analysis. J. Neurosci. Methods.

