# Supplementary material for: Superior frontal regions reflect the dynamics of task engagement and theta band-related control processes in time-on task effects
Source: Sci Rep. 2022 Jan 17;12:846. doi: 10.1038/s41598-022-04972-y (PMC8763946; doi:10.1038/s41598-022-04972-y)
Supplement: Supplementary file 1 — Supplementary Information. [file 41598_2022_4972_MOESM1_ESM.pdf]

Supplementary Materials

Superior frontal regions reflect the dynamics of task engagement and theta band-related control processes in time-on task effects

Shijing Yu, Moritz Mückschel, Christian Beste

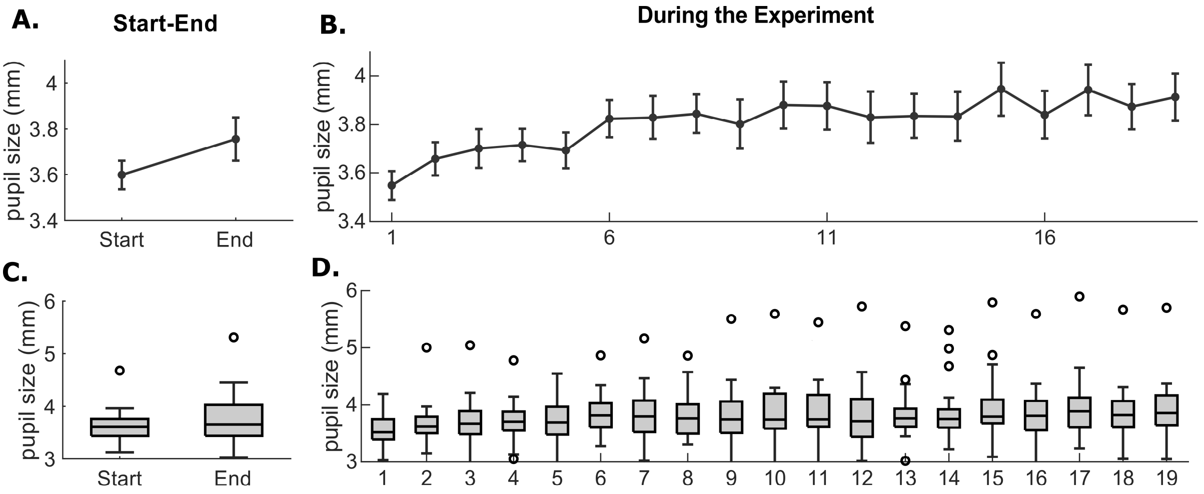

Figure 1. Pupil diameter baseline in fixation periods. Pupil diameters in baseline level/resting states were extracted from N=26 subjects, 1 subject was excluded due to incomplete data. A shows the average and SEM of pupil size before and after the experiment. C and D are boxplots respectively corresponding to A and B.

Table.1 Behavioral performance

	Hit rate (%)		*Correct rejection rate (%)*		*RT(milliseconds)*	
	Go 30°	*Go 150°*	*Nogo 30°*	*Nogo 150°*	*Go 30°*	*Go 150°*
Session 1	94.23 ± 7.68	87.89 ± 12.72	93.46 ± 7.61	86.89 ± 12.89	587.08±55.37	689.58±61.16
Session 2	93.27 ± 9.43	90.65 ± 11.11	93.91 ± 5.17	90.25 ± 10.90	580.47±56.92	660.03±63.86
Session 3	91.89 ± 10.81	88.23 ± 11.99	93.27 ± 5.88	90.27 ± 12.06	589.25±54.22	662.56±56.18
Session 4	90.67 ± 8.46	86.60 ± 11.01	91.25 ± 7.61	89.15 ± 10.15	602.49±62.07	671.72±61.14

Table.2 Peak size of pupil diameter (in millimeter)

	Go 30°	*Go 150°*	*Nogo 30°*	*Nogo 150°*
Session 1	.062 ± .042	.078 ± .059	.026 ± .040	.041 ± .049
Session 2	.059 ± .040	.075 ± .054	.026 ± .039	.033 ± .048
Session 3	.056 ± .035	.062 ± .042	.022 ± .037	.025 ± .042
Session 4	.050 ± .032	.054 ± .037	.018 ± .032	.021 ± .039

Table.3 Latency of peak pupil diameter (in milliseconds)

	Go 30°	*Go 150°*	*Nogo 30°*	*Nogo 150°*
Session 1	1.164 ± .307	1.218 ± .334	1.027 ± .426	1.128 ± .437
Session 2	1.176 ± .322	1.213 ± .307	1.026 ± .382	1.059 ± .413
Session 3	1.209 ± .261	1.257 ± .283	1.091 ± .359	1.097 ± .390
Session 4	1.247 ± .256	1.268 ± .262	1.155 ± .363	1.197 ± .420
